# HIV-1 Phenotypic Reverse Transcriptase Inhibitor Drug Resistance Test Interpretation Is Not Dependent on the Subtype of the Virus Backbone

**DOI:** 10.1371/journal.pone.0034708

**Published:** 2012-04-09

**Authors:** Michelle Bronze, Kim Steegen, Carole L. Wallis, Hans De Wolf, Maria A. Papathanasopoulos, Margriet Van Houtte, Wendy S. Stevens, Tobias Rinke de Wit, Lieven J. Stuyver, on behalf of the ART-A Consortium

**Affiliations:** 1 Department of Molecular Medicine and Haematology, University of the Witwatersrand, Johannesburg, South Africa; 2 Virco BVBA, Beerse, Belgium; 3 Center for Poverty-related Communicable Diseases (CPCD), PharmAccess Foundation, Academic Medical Center, Amsterdam, The Netherlands; INSERM, France

## Abstract

To date, the majority of HIV-1 phenotypic resistance testing has been performed with subtype B virus backbones (e.g. HXB2). However, the relevance of using this backbone to determine resistance in non-subtype B HIV-1 viruses still needs to be assessed. From 114 HIV-1 subtype C clinical samples (36 ARV-naïve, 78 ARV-exposed), *pol* amplicons were produced and analyzed for phenotypic resistance using both a subtype B- and C-backbone in which the *pol* fragment was deleted. Phenotypic resistance was assessed in resulting recombinant virus stocks (RVS) for a series of antiretroviral drugs (ARV's) and expressed as fold change (FC), yielding 1660 FC comparisons. These Antivirogram® derived FC values were categorized as having resistant or sensitive susceptibility based on biological cut-off values (BCOs). The concordance between resistance calls obtained for the same clinical sample but derived from two different backbones (i.e. B and C) accounted for 86.1% (1429/1660) of the FC comparisons. However, when taking the assay variability into account, 95.8% (1590/1660) of the phenotypic data could be considered as being concordant with respect to their resistance call. No difference in the capacity to detect resistance associated with M184V, K103N and V106M mutations was noted between the two backbones. The following was concluded: (i) A high level of concordance was shown between the two backbone phenotypic resistance profiles; (ii) Assay variability is largely responsible for discordant results (i.e. for FC values close to BCO); (iii) Confidence intervals should be given around the BCO's, when assessing resistance in HIV-1 subtype C; (iv) No systematic resistance under- or overcalling of subtype C amplicons in the B-backbone was observed; (v) Virus backbone subtype sequence variability outside the *pol* region does not contribute to phenotypic FC values. In conclusion the HXB2 virus backbone remains an acceptable vector for phenotyping HIV-1 subtype C *pol* amplicons.

## Introduction

Within the past decade, access to antiretroviral therapy (ART) for HIV-1 infection has increased exponentially in low- and middle-income countries. More than six million people were receiving highly active antiretroviral therapy (HAART) in these countries at the end of 2010, as compared to just 400 000 at the end of 2003 [1]. However, a major hurdle to sustainable, successful ART is the inevitable emergence of HIV-1 drug resistance. In addition, inadequate resources and health care infrastructure in these regions, as well as the introduction of ART, can create conditions for the accelerated development of HIV-1 resistance to antiretrovirals (ARVs) [2], further compromising the patients' future treatment options. Hunt *et al.* (2011) showed that an average of 34% of South African children under the age of 24 months had developed non-nucleoside reverse transcriptase inhibitor (NNRTI) resistance, in particular the Y181C mutation, when they were previously exposed to single dose nevirapine (sdNVP) [3].

HIV-1 transmitted drug resistance mutations (TDRMs) were evaluated in recently infected individuals from some East and Southern African countries, and showed a 5.0 and 5.6% prevalence respectively [4], [5]. Hamers *et al*. (2010) [6] found that HIV-1 drug resistance mutations were present in 6% of patients initiating ART in Lusaka, Zambia. Levels of transmitted resistance have been shown to be 8.6% in Kampala, Uganda [7]. In light of these findings, focus should be placed on optimal frequency of both viral load testing and appropriate antiretroviral drug resistance testing.

HIV-1 ARV drug resistance is usually measured by genotypic testing. It still remains an expensive test and is not yet an option for individual patient management in resource poor settings, but is a vital tool for resistance surveillance of large-scale HIV treatment programs. During genotypic resistance testing, the nucleotide sequence of specific HIV-1 genes, which are responsible for ARV drug resistance, are determined and fed into a predictive algorithm, describing the susceptibility to a range of ARVs. The *pol* region is sequenced when the drug therapies of the patient contain nucleoside reverse transcriptase inhibitors (NRTIs), non-nucleoside reverse transcriptase inhibitors (NNRTIs), and protease inhibitors (PIs). Most of the algorithms for predicting drug resistance are based on data derived from *in vivo* (clinical outcome data) *or in vitro* phenotypic testing of subtype B virus (virco®TYPE and PhenoSense® GT).

However, HIV-1 subtype C accounts for over 48% of all global infections, and is the predominant circulating subtype amongst the heterosexual population in sub-Saharan Africa [8]. The prevalence of HIV-1 subtype C resistance amongst patients failing first-line HAART has been shown to be 82% in the South African public sector [9] and in a study by Murphy *et al.* 2010 [10], it was noted that 87% of patients on HAART for 12 months had developed at least one resistance mutation.

In contrast to HIV-1 genotype resistance testing, phenotyping is an *in vitro* assay, which measures the ability of a virus to replicate in the presence of a drug. Currently, most available phenotyping assays are based on recombining patient-derived sequences into a subtype B backbone deleted for the corresponding patient sequences. HIV-1 phenotyping is considered to be the gold standard in resistance testing, although it is has only been performed, using subtype B backbones. Phenotyping is not a tool that could be adapted to resource limited settings due to its high cost, infrastructural requirements, and technical skill needed. Until recently, it remained, however, unclear whether a recombinant virus assay using a subtype B backbone would correctly measure drug resistance when the patient-derived sequences are of subtype C. The Antivirogram® assay [11] recombines patient-derived PR and RT sequences into an HIV-1 subtype B (HXB2) backbone deleted for these sequences [12]. Nauwelaers *et al.* (2011) [13] constructed an HIV-1 subtype C-backbone within the Antivirogram® assay setting, and tested eight subtype C samples on a clonal level within both an HIV-1 subtype B- and C- backbone. Resistance profiles generated were similar in both backbones. The present study is an extension of the work by Nauwelaers *et al. (2011)*, comparing population-based phenotypic HIV-1 drug resistance profiles of subtype C gag-protease-reverse transcriptase (GPRT) sequences generated using a subtype B- and C-backbone. The aim was to assess whether an HIV-1 subtype B- backbone could be used with a high level of confidence to phenotype subtype C samples.

## Materials and Methods

### 1. Ethics Statement

Ethical clearance for this study was obtained and approved for by the Human Research Ethics Committee (HREC) at the University of the Witwatersrand (Clearance Number M090688), and for the PASER-M cohort from the Academic Medical Center Institutional Review Board and the University of Zambia Research Ethics Committee. Informed consent was obtained for samples from the PASER-M cohort, but not for those from the University of the Witwatersrand, as it was not required for the ethical approval obtained from the HREC. The data derived from this work was for research and development purposes and for method validation only. According to HREC policies, for this type of study, these specimens did not require patient enrollment or informed consent, and a waiver was hence granted.

### 2. Patient samples used in this study

Plasma samples received for routine population-based HIV-1 drug resistance genotyping were analyzed with the genotyping assay described by Wallis *et al*. (2010) [14]. A total of 265 samples were used for further phenotypic testing. Two hundred and fifteen (215) samples were obtained from treatment-experienced patients attending clinics in the public sector in Johannesburg, South Africa, and selected specifically for the presence of HIV-1 ARV drug resistance. Fifty (50) treatment-naïve samples were selected from the PASER-M cohort [15], based on available genotypic information.

### 3. Viral RNA Extraction

Viral RNA was isolated from all patient plasma samples using the MagNA pure LC Total Nucleic Acid Isolation Kit (Roche, Mannheim, Germany) with a sample input of 200 µl, and a 50 µl elution volume, as per manufacturer's instructions. Viral RNA from recombinant virus stocks (RVS) was extracted using the QIAamp Virus BioRobot MDx kit (Qiagen, Belgium) or the NucliSENS easyMAG (bioMérieux Inc, Belgium), starting with an input volume of 600 µl and eluting in 25 µl, as per manufacturer's instructions.

### 4. Gag-Protease-Reverse Transcriptase (GPRT) amplification and sequencing

Samples were analysed in a two-step approach. First, an RT-PCR amplification protocol [14] was used to amplify a 1.5 kb *pol* fragment. This is later referred to as protocol 1. The resulting HIV-1 genotype was used to select for resistant samples for this study. Secondly, a 1.9 kb GPRT fragment was amplified (One-Step SuperscriptIII High Fidelity, Invitrogen, CA, USA) using the “3′-RT” and “5′-OUT” primers [13], with a 10 µl RNA input in a total volume of 35 µl. Nested PCR was performed using the Expand High Fidelity PCR System (Roche Diagnostics GmbH, Manheim, Germany), with 8 µl of first round amplicon and primers 3′IN, and 5′IN in a final volume of 100 µl [13] resulting in a final amplicon encompassing nucleotides 2012 to 3879 in *pol* (according to HXB2 numbering – genbank: AF033829). This second protocol is further referred to as protocol 2. Amplification products were analyzed by 1% agarose gel electrophoresis, and amplicons were purified using the QIAquick PCR Purification Kit (Qiagen, Hilden, Germany) following manufacturer's instructions.

Sequencing was performed using the Big Dye Terminator Cycle Sequencing Kit v3.1 (Applied Biosystems, CA, USA) as described previously [13]. Cycle sequencing purification was performed using the DyeEX (Qiagen, Hilden, Germany) purification kit, according to manufacturer guidelines. The ABI3730 XL (Applied Biosystems, CA, USA) performed the sequence detection and analysis was performed using the Sequencher v.4.5 software (Gene Codes Corporation, MI, USA).

### 5. Genotypic Analysis

Sequence data generated from first-step analysis was submitted to the Stanford University HIV drug resistance database [16] to generate an ARV drug resistance profile and subtype. Based on the ARV drug resistance profiles, resistant samples were selected for the GPRT amplification, and subsequent phenotypic drug resistance analysis. The second-step (GPRT amplicon) sequence data was submitted to the Stanford University HIV drug resistance database.

### 6. In vitro phenotypic ARV drug resistance analysis

Recombinant virus stocks (RVS) were generated through homologous recombination of each of the GPRT amplicons into subtype B- and subtype C- backbones for use in the Antivirogram® assay, as described by Hertogs *et al*. (1998) [12]. Generation of the subtype C recombinant viruses was performed in MT4/eGFP cells [13], whereas subtype B recombinant viruses were generated in MT4 cell lines, respectively using eGFP expression and Cytopathic Effect (CPE) scoring, respectively to monitor adequate viral growth. One hundred (100) µl of harvested subtype B and C recombinant viruses were then titrated in MT4/eGFP cells. A panel of 18 ARV drugs (see below) was used in the antiviral experiment to establish the resistance profile of the RVS.

**Figure 1:**
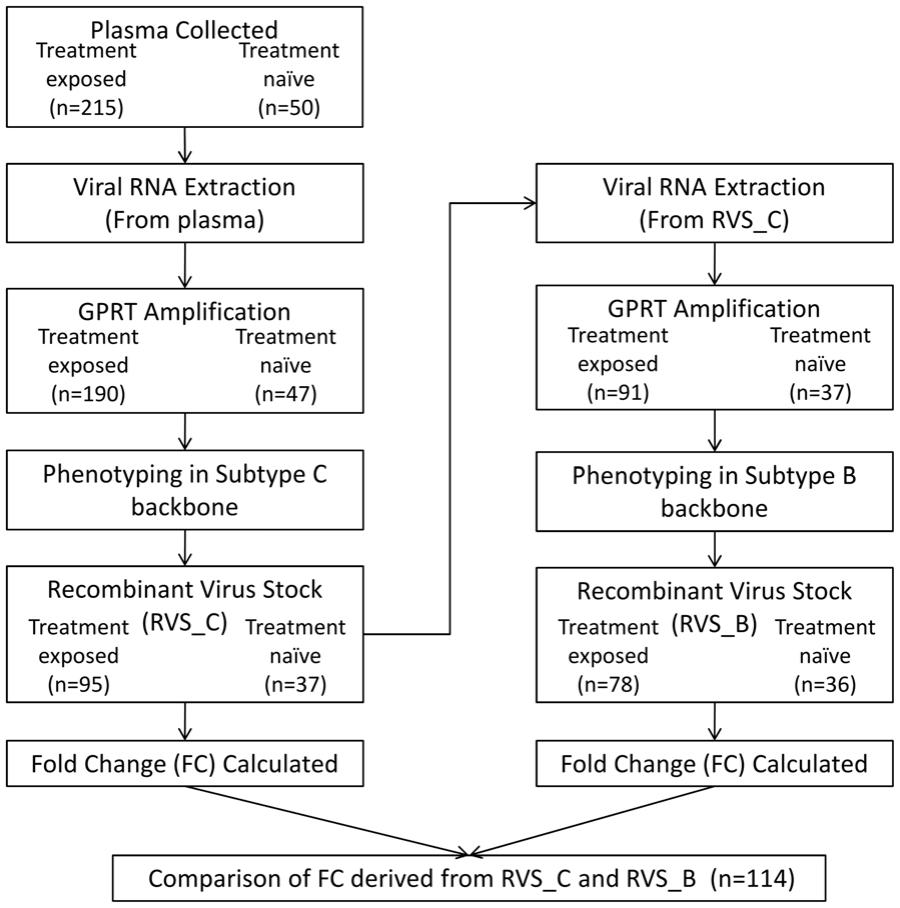
Process flow diagram for phenotyping procedure within subtype B- and C- backbones.

Each GPRT amplicon was initially used to generate a full subtype C recombinant virus (RVS_C) which was phenotyped in the Antivirogram® assay. After phenotyping, the GPRT region of the recombinant subtype C- backbone virus was PCR amplified as described above, and the resulting amplicon was genotyped and used to generate a recombinant subtype B- backbone virus. The recombinant subtype B- backbone virus (RVS_B) was subsequently phenotyped, followed by PCR amplification and genotyping of the GPRT region. The genotyping of the GPRT region was performed at all three time points (plasma, RVS-C and RVS-B) to ensure that the genetic background of the recombinant viruses was identical throughout the phenotyping experiments (Figure 1). This strategy was undertaken to first phenotype in a C-backbone because C-backbone phenotyping took 10 to 23 days to harvest virus, whilst only 5 to 10 days in a B-backbone. During this extended time to harvest with the C-backbone, it was a concern that there may have been some significant viral evolution during that time, therefore the RVS_C that was harvested was used as input into the B-backbone phenotyping.

Wild type subtype B (HXB2) and C [13] cell line adapted viruses were used as reference viruses for the subtype B- and C- backbone experiments, respectively. All 50% inhibitory concentration (IC_50_) values were calculated from 8 readings for each recombinant virus against the different ARV drugs. Fold changes (FC) were calculated by dividing these IC_50_ values by the IC_50_ of the appropriate reference virus. Viruses were characterized as being susceptible or resistant based on pre-established biological cut-off values (BCOs) [17].

ARV drugs and drug concentrations (µM) used in the antiviral experiment included lamivudine (3TC; 0.12 to 31.25), zidovudine (AZT; 4.88 10^−3^ to 1.25), stavudine (D4T; 0.24 to 62.50), didanosine (DDI; 0.24 to 62.5), abacavir (ABC; 0.49 to 125), emtricitabine (FTC; 7.63 10^−4^ to 5.00), tenofovir (TDF; 0.12 to 31.25), nevirapine (NVP; 4.88 10^−3^ to 1.25), efavirenz (EFV; 1.53 10^−4^ to 10.00), etravirine (ETR; 3.82 10^−5^ to 2.50), indinavir (IDV; 4.88 10^−3^ to 1.25), fosamprenavir (APV; 4.88 10^−3^ to 1.25), atazanavir (ATV; 0.48 10^−5^ to 0.31), saquinavir (SQV; 0.48 10^−5^ to 0.31), darunavir (DRV; 3.82 10^−5^ to 2.50), lopinavir (LPV; 0.24 10^−5^ to 0.16), and tipranavir (TPV; 0.02 to 5.00).

### 7. Statistical Analysis

Possible inter-batch IC_50_ differences between the wild-type viruses, HXB2 and wildtype C were investigated for each ARV by means of an analysis of variance (ANOVA). This was done by comparing the IC_50_'s of all drugs repeatedly tested throughout 10 batches of experiments.

The comparison of resistance and susceptibility call rates between the B- and C-backbone phenotyping were assessed by calculating the sensitivity and specificity of the C-backbone phenotype as compared to the B-backbone phenotype (regarded as the gold standard). Sensitivity was calculated as (true sensitive/(true sensitive + false resistant)×100) and specificity was calculated as (true resistant/(true resistant + false sensitive)×100). Hence, sensitivity measures the proportion of sensitive calls, which are correctly identified, while specificity measures the proportion of resistant calls, which are correctly identified.

Fold Change comparisons for which a discordant resistance call was observed between B-backbone and C-backbone were further assessed within the context of the intra-assay variability. Initially, all FC values were “normalized” against BCO's for the various drugs, by subtracting the relevant ARV's BCO from that FC value. Hence, all FC values, regardless of the ARV that was tested, could be compared collectively. FC values that gave the same resistance call, regardless of their backbone of descent were subtracted and used to set the acceptable FC variability limits between both backbones. The mean +/−2 standard deviations (2SD) was calculated for the concordant FC comparisons (susceptible and resistant viruses separately). The difference of log FC values of the discordant data points were calculated, and plotted on a Bland-Altman plot using the mean +/−2SD derived from the concordant samples as cut-offs. Any values outside these cut-offs were considered to not fall within these acceptable limits of assay variation, and hence truly discordant data points.

In addition, the capacity of the two backbones to detect resistance caused by predominant subtype C ARV drug resistance associated mutations was also assessed using Receiver of Operator Characteristics (ROC) [18].

## Results

### 1. Generating recombinant virus stocks from subtype C- and B- viruses

A total of 265 clinical isolates were available for testing, 215 from ARV experienced patients, and 50 from therapy naïve patients. In the protocol 2 amplification procedure [13], 237 GPRT amplicon were obtained, 190 and 47 from treatment-experienced and naïve patients respectively. The 237 GPRT amplicons were used to generate 132 subtype-C recombinant virus stocks (RVS-C), which were subsequently GPRT amplified, and recombined with a subtype B- backbone. One-hundred and fourteen (114) subtype B RVS were generated. These RVS were GPRT sequenced and compared. Only RSV-C and RSV-B strains with identical genotypic analysis were included in further analysis. Finally, 114 paired B- and C- backbone recombinant viruses (78 from treatment-exposed +36 from treatment-naive) were retained for further analysis. The process flow phenotypic testing is given in Figure 1.

### 2. Genotypic analysis of the 114 sequences

The HIV-1 drug resistance mutation profiles [19] for the treatment-exposed group (n = 78) were analyzed. The most prevalent mutations in this dataset included: K103N (n = 30; 38.5%), M184V (n = 26; 33.3%), T215Y (n = 13; 16.7%), T215F (n = 8; 10.3%), M41L (n = 9; 11.5%), V106M (n = 8; 10.3%), D67N (n = 9; 11.5%), V108I (n = 8; 10.3%). The treatment naïve group (n = 36) had no ARV drug resistance mutations.

### 3. Evaluation of the subtype C virus assay variability in phenotypic testing

Prior to the IC _50_ –values determination of the 114 RVS-C and RVS-B preparations, assay variability for the subtype C Antivirogram® assay was determined. The wild-type virus IC_50_ values were compared over the course of all experiment batches (n = 10) performed and for each drug tested, to determine assay variability. No difference in the variance of the IC_50_ –values of the wild-type viruses was noted amongst all drugs tested for the 10 experiments performed (p = 0.41).

### 4. Analysis of the phenotypic resistance determinations on 114 RVS-B and RVS-C strains

FC calculations, using respective wild-type viruses, were performed to determine whether an RVS was sensitive or resistant to a specific ARV. A summary of the subtype B- and C- backbone phenotyping resistance profiles is shown in Table 1. Not all 114 paired comparisons were obtained for every ARV. For example, for AZT a total of 82 of the expected 114 comparisons were obtained, resulting in an AVE success rate of 71.9% for AZT. Using the Antivirogram® BCO's [17], the FC values for the RVS-B (gold standard) were designated as being either sensitive (n = 1272) or resistant (n = 378) to a particular ARV. Using the same BCO's as for the B- backbone, the C- backbone phenotype resistance calls were determined as sensitive (n = 1192) and resistant (n = 458). A total of 1650 paired subtype B- and C- backbone derived FC comparisons were obtained, with an overall AVE success rate for all drugs of 85.1%.

**Table 1:** Comparison between HIV-1 subtype B and C backbone phenotyping resistance profiles.

ARV	BCO (FC)	AVE success rate (%)	B Backbone Phenotype		C Backbone Phenotype		Total pairedComparisons (n)	SENSITIVITY (%)	SPECIFICITY (%)
			SENSITIVE (n)	RESISTANT (n)	SENSITIVE (n)	RESISTANT (n)			
AZT	2.5	71.9	74	8	72	10	82	97.3	100.0
3TC	2.1	76.3	45	42	51	36	87	91.1	76.2
DDI	2.3	95.6	73	36	61	48	109	69.9	72.2
D4T	2.2	79.8	89	2	82	9	91	92.1	100.0
ABC	2.3	96.5	80	30	66	44	110	78.8	90.0
FTC	3.1	85.1	52	45	46	51	97	84.6	95.6
TFV	2.2	96.5	101	9	89	21	110	83.2	44.4
**NRTI**		**86.0**	**514**	**172**	**467**	**219**	**686**	**85.3**	**82.6**
NVP	6.0	93.0	52	54	46	60	106	86.5	98.1
EFV	3.3	85.1	42	55	36	61	97	81.0	96.4
ETR	3.2	86.8	79	20	67	32	99	77.2	70.0
**NNRTI**		**88.3**	**173**	**129**	**149**	**153**	**302**	**81.6**	**88.2**
IDV	2.3	74.6	76	9	77	8	85	98.7	77.8
SQV	1.8	74.6	82	3	77	8	85	92.7	66.7
APV	2.2	89.5	90	12	93	9	102	98.9	66.7
LPV	1.6	81.6	73	20	68	25	93	78.1	45.0
ATV	2.1	86.0	86	12	82	16	98	91.9	75.0
TPV	1.7	96.5	94	16	96	14	110	91.5	37.5
DRV	2.0	78.1	84	5	83	6	89	94.0	20.0
**PI**		**83.0**	**585**	**77**	**576**	**86**	**662**	**92.3**	**55.5**
**All_Drugs**		**85.1**	**1272**	**378**	**1192**	**458**	**1650**	**87.5**	**72.4**

ARV abbreviation: lamivudine(3TC), zidovudine (AZT), stavudine (D4T), didanosine (DDI), abacavir (ABC), emtricitabine (FTC), tenofovir (TFV), nevirapine (NVP), efavirenz (EFV), etravirine (ETR), indinavir (IDV), fosamprenavir (APV), atazanavir (ATV), saquinavir (SQV), darunavir (DRV), lopinavir (LPV), tipronavir (TPV). Sensitivity was calculated as (true sensitive/(true sensitive + false resistant)×100) and specificity was calculated as (true resistant/(true resistant + false sensitive)×100). BCO: Biological Cut-off. AVE: Antiviral Experiment. FC: Fold Change.

**Figure 2:**
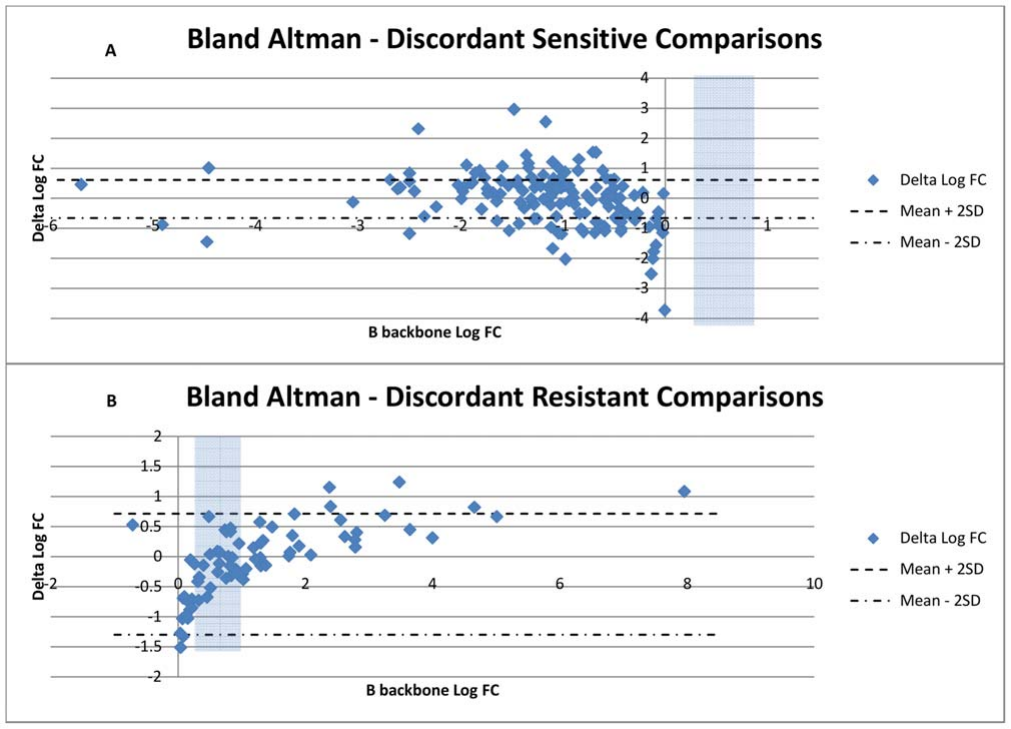
Plots showing discordant phenotypic resistance comparisons, and variation around the BCO. The FC of the B backbone derived viruses (assumed to be the gold standard) are shown on the x-axis, and the difference in log FC values (B backbone FC – C backbone FC) on the y-axis. The shaded region on the plots is the region wherein the log FC biological cut-offs lie for the 18 ARVs tested. The mean +2SD and mean −2SD are drawn in to illustrate the natural variation around the BCO. Any point s found above the mean +2SD or below the mean −2SD are considered to be truly discordant. Figure 2A is a plot of comparisons within the B'sens/C'res group. Figure 2B is a plot of comparisons within the B'res/C'sens group.

Resistance and susceptibility call rates were compared to obtain sensitivities and specificities of the C- backbone phenotyping (Table 1). Sensitivity ranged from 69.9% to 89.9% (overall mean 87.5%), and specificity from 20.0% to 100.0% (overall mean 72.4%). A low sensitivity depicts that the C- backbone is over-calling resistance, and a low specificity means that the C- backbone is under-calling resistance. The observed resistance call discordances were further assessed for their relationship to genotypic predictions (Table 2), and biological variation, which may affect resistance calling in those samples with FC values close to the BCO (Figure 2).

**Table 2:** Stanford University HIV Drug Resistance Database Resistance Profiles of Phenotypic Comparisons.

Phenotypecategory	Stanford HIVdb ARV Drug Resistance Profiles				
	Total (n)	Sensitive + PotentialLow Resistance (n)	LowLevel Resistance (n)	IntermediateLevel Resistance (n)	HighLevel Resistance (n)
B'sens/C'sens†	**1118**	NP	NP	NP	NP
B'res/C'res‡	**304**	NP	NP	NP	NP
B'sens/C'res*	154	90	25	25	14
B'res/C'sens**	74	62	6	6	0

NP: Comparison not performed

Stanford HIVdb genotypic data not shown for concordant comparison, as this was only done to try ascertain if one backbone's phenotype was consistently miscalling (with reference to genotype).

† B'sens/C'sens are those comparisons which were sensitive in both the B and C backbone.

‡ B'res/C'res‡are those comparisons which were resistant in both the B and C backbone.

* B'sens/C'res are those comparisons which were sensitive in the B backbone and resistant in the C backbone.

** B'res/C'sens are those comparisons which were resistant in the B backbone and sensitive in the C backbone.

### 5. Concordance and discordance analysis

The concordant and discordant data points are summarized in Table 2. Firstly, 1118 data points were called sensitive and 304 called resistant in both backbones. This resulted in 86.2% (1422/1650) concordant data points. Secondly, comparisons which were sensitive in the B- backbone and resistant in the C- backbone (B'sens/C'res) (n = 154) were 58.4% (90/154) concordant with a sensitive genotypic call in the genotypic interpretations from the Stanford HIV Drug Resistance database [16]. The comparisons which were resistant in the B- backbone and sensitive in the C- backbone (B'res/C'sens) were 16.2% (12/74) concordant with a resistant genotypic interpretation.

### 6. Analysis of the observed variability of FC values between backbones

To ascertain the acceptable variability of FC values generated in both backbones, the mean FC difference +/−2SD of the concordant samples were calculated. This was plotted on a Bland Altman plot, to visualize truly discordant B- and C- backbone comparisons, with the log FC of B- backbone derived data on the x-axis, and the difference between the B- and C- backbone derived log FC's on the y-axis (Figure 2). Under these settings, 95.8% (i.e. 1590 from 1660 comparisons) of the phenotypic data derived from subtype B- backbone viruses had a concordant resistance call with subtype C- backbone derived data. Sixty-three (63) and 7 data points lie outside of the mean ± 2SD for the B'sens/C'res (Figure 2A) and B'res/C'sens (Figure 2B) data, respectively. Collectively, these 70 data points were considered discordant data points. The 70 discordant comparisons were predominantly from samples with a resistant genotype, who were treatment experienced (60/70), with 10 out of the 70 being from sensitive and treatment naïve samples.

### 7. ROC curves

Finally, the capacity of the different backbone phenotyping to predict ARV drug resistance associated with the M184V, K103N and V106M mutations was assessed. Only these 3 mutations were assessed, as other mutations in this sample set had too few observations for meaningful data interpretation. Table 3 summarizes ROC curve analyses, which demonstrates the tradeoff between sensitivity and specificity for the subtype B- and C- backbone based phenotyping assays in detecting decreased sensitivity caused by M184V, K103N and V106M mutations. The tests of differences between the areas under the empiric ROC curves show that regardless of the range of BCOs, the two backbones report the same detection sensitivities for ARV drug resistance to these 3 mutations.

**Table 3:** Receiver of Operator Characteristic (ROC) statistics for 3 common HIV-1 subtype C resistance mutations.

Resistance Mutation	Antiretroviral Drug	Number of Observations		ROC Curve Statistics		
		Withresistance mutation	Without anyresistance mutations	Difference(B–C)	CI	P-value
M184V	3TC	27	33	0.01	−0.21, 0.18	0.896
	FTC	39	27	0.02	−0.05, 0.01	0.218
K103N	NVP	37	31	0.01	−0.04, 0.01	0.278
	EFV	34	29	0.0009	−0.003, 0.001	0.480
V106M	NVP	14	31	0.14	−0.39, 0.11	0.272
	EFV	12	29	0.17	−0.43, 0.08	0.187

## Discussion

The emergence of ARV drug resistance in HIV-1 infected patients requires that clinicians make informed decisions when selecting the next ARV regimen based on genotypic and/or phenotypic drug resistance testing. However, based on its cost and logistic requirements, genotyping may not yet be an HIV-1 drug resistance monitoring tool in resource limited settings. Nonetheless, this methodology can certainly be used in monitoring HIV resistance on a population-based level at selected sites in Africa. The current study is primarily meant to ensure that the genotyping data generated through such monitoring programs for Africa are supported by phenotyping as a gold standard. This study evaluated the feasibility of using a phenotypic assay (Antivirogram®) based on a subtype B- backbone for resistance testing of subtype C infected patient samples.

Choe *et al.* (2007) [20] reported that the interpretation of ARV drug susceptibility using the Phenosense® phenotypic assay was not dependent on the subtype of the backbone vector (B vs. C). Furthermore, comparative analyses of commercially available phenotypic assays, Antivirogram® with PhenoSense® [21], and the virco®TYPE HIV-1 (*virtual* **Phenotype**) with PhenoSense® [22] found that results correlate well, despite the use of different testing strategies. These two assays amplify the 3′ part of *gag*, including the p7/p1 and p1/p6 cleavage sites, the entire *protease*, and most of *RT* (Antivirogram®: RT amino acids 1–400 and PhenoSense®: RT amino acids 1–311) [12]; [23]. With the minor difference in the length of RT sequence (aa 311–400) used in these two assays, it is thus expected that the subtype of the backbone used in the Antivirogram® assay should also not impact on the interpretation of ARV drug susceptibility of non-B subtypes. Although the IAS guidelines [19] suggest that no known resistance mutations appear between amino acids 311 and 400, the virco®TYPE HIV-1 algorithm [24] and the Stanford University drug resistance database [16] have listings of resistance mutations within this region. No difference in these assays are expected, however, since Steegen *et al.* [24] showed that using a shortened RT sequence (aa41–238) for genotyping, still gave comparable genotypic resistance results as sequencing a full RT.

Amplification of the region encompassing *gag-*PR-RT was necessary for recombination with the Antivirogram® subtype B- and C- backbone [13]. Unlike Nauwelaers *et al*. (2011) [13], who used a clonal phenotyping approach, this work used population-based phenotyping, which depicts what would happen in a clinical setting. This population-based approach is preferred due to ease of use in comparison to the clonal approach.

Of the 133 amplicons initially phenotyped in a C- backbone, 114 paired subtype B and C phenotypes were obtained. During the phenotyping process, some of the transfections failed to yield recombinant viruses especially in the C- backbone. Often it was noted that even though adequate virus was scored with eGFP scoring for the C- backbone, when titrated, the desired yield of virus was not obtained. The standard CPE scoring is not an option for C-backbone viruses, as subtype C viruses do not produce CPE in these cell types [25], [13], and therefore other alternatives were required (eg. eGFP scoring using MT4/EGFP cells). A suggestion is that a more direct measurement of virus concentration should be used for the scoring of these viruses (eg. p24).

With 114 paired B- and C-phenotype comparisons, and 17 drugs tested, the expected number of FC comparisons would be 1938. Only 1650 comparisons were obtained however, with an overall AVE success rate of 85.1%. A lowered AVE success rate was generally noted to be a result of failure to meet the quality control criteria set for Antivirogram® analyses of either a B- or C- backbone derived IC_50_ reading. Fluorescent pixel intensity readings for subtype C- backbone derived viruses were often too high for accurate IC_50_ calculations to be made. This could be attributed to the different scoring method used for C-backbone viruses or altered replication capacities of the different virus subtypes, but was not investigated further in this study.

Resistance and susceptibility calls were compared between the phenotypes derived from the two backbones, as one of the primary objectives of the study was to analyze the concordance of results by phenotypic category because of the direct implications for patient management. Sensitivity and specificity was calculated for each ARV drug tested, using the subtype B- backbone as the gold standard, and hence subtype B derived BCO's as the ubiquitous cut-offs. An 87.9% (average sensitivity) of susceptible calls (FC<BCO) were correctly identified in the C- backbone. For some drugs, like ddI, ABC, ETR and APV, with sensitivities of less than 80%, it appears that the C- backbone is over-reading resistance as compared to B-backbone results. In terms of specificity, a 72.4% average is noted overall, with markedly low specificities observed within the PI group. The interpretation of which, is that there is some over-calling of resistance occurring in the B- backbone, particularly within the PI group. This may be a result of there being an insufficient amount of data illustrating HIV-1 subtype C drug resistance to PIs in this study, as PIs are mainly part of the second-line regimen in South Africa, which has resulted in few patients developing PI resistance.

A high number of concordant resistance calls were reported, with 1118 comparisons within the B'sens/C'sens category, and 304 within the B'res/C'res category, thus accounting for 86.2% (1422/1650) of comparisons tested. The discordant resistance calls resulting were compared with genotypic profiles as per the Stanford University drug resistance database [16] to investigate which backbone is theoretically giving the correct call (Table 2). Ultimately, subtype B backbone phenotyping was being assessed for use with subtype C specimens. In the following discordant B- and C-backbone resistant calls, a comparison was made of how the B-backbone resistance calls fare with a genotypic algorithm. Data shows that for discordant comparisons, the sensitive B- backbone phenotype agreed with genotypic sensitive calls for 58.4% (5.5% (90/1650) of resistance over-calling) of these cases, and only 16.2% of resistant B- backbone phenotype corresponded to the resistant genotype. The implications of the 16.2% correct calling of resistance in the B backbone was that, in terms of the genotypic prediction algorithm the B- backbone had a 0.7% rate of under-calling resistance in these particular cases. Notably, these discordant samples only account for 13.8% (228/1650) of total comparisons made. These discordant comparisons were quite diverse, with no trends being noted in terms of resistance mutations and/or ARV resistance profiles affected, hence results could not be further elaborated upon. Nonetheless, the clinical implications of the under-calling of resistance would be that patients would remain on a failing drug regimen whilst accumulating more resistance. The over-calling of resistance would mean that patients would be switched too early onto second-line regimens.

Restrictions encountered with these analyses are that only subtype B derived BCO's are available, and these discrepancies may be a result of using inappropriate BCO's for subtype C- backbone viruses. The above analyses are also reliant on a single BCO value, not taking into account any variation around that cut-off nor of the assay variation. If a subtype-specific C-backbone is to be used, it would need to be investigated whether subtype-specific BCO's would need to be derived. Phenotypic output throughout various assays is shown to have expected inter- and intra- assay variability due to the nature of this *in vitro* assay [20], [21], [26], [27]. By indirectly taking these confounding factors into account (i.e. Figure 2), the truly discordant comparisons were targeted (outside the mean +/−2SD range). Collectively, it was calculated that 95.8% (1590/1660) of all phenotypic data derived from the subtype B- backbone virus had a concordant resistance profile with that of the subtype C- backbone derived viruses. Of the 70 discordant values found, only 10 comparisons were from treatment naïve (sensitive virus as per Stanford drug resistance database predictions), re-iterating that the B- and C- backbone assay variability is similar. Similar results were noted in a study by Choe *et al*. (2007) [20], who performed an analogous experiment within the PhenoSense® assay, showing a concordance of 95.8% of pair wise FC value comparisons across all drugs for all subtype C viruses tested in B- and C- backbone.

Another minor restriction of this analysis is that the majority of recombinant viruses carried the M184V, the K103N and the V106M mutations, which provide high-level resistance to 3TC and FTC with M184V or EFV and NVP with K103N and V106M. The virus backbone in such cases may theoretically have a decreased, if any, impact on the phenotypic result. In the context of the sample set used in this analysis, the prevalence of other mutations considered to not be classed as high-level resistance were not present in high amounts, and as such this could not be further studied.

An assessment of the sensitivities of these two backbones to measuring resistance in the presence of the M184V, K103N, or V106M mutation was performed. These particular mutations were selected for this analysis, in the context that subtype C viruses are under investigation in these experiments, and these mutations have previously been shown to be some of the most prevalent drug resistance mutations in subtype C treatment failures [10]. It would have been of interest to look at the K65R and thymidine analogue mutations (TAMs), but there were insufficient data points available for appropriate ROC analysis to be performed. ROC curves were plotted (data shown in Table 3), and no statistical differences noted with any of these 3 mutations. The detection sensitivity of both backbones was equal in measuring resistance to these prominent drug resistance mutations.

No systematic resistance under- or overcalling of the subtype C amplicons in the B- backbone phenotyping was noted. It appears that the virus backbone susceptibility outside of the GPRT region does not contribute to any changes in phenotypic FC values. The practical question being considered in this work is whether or not it is reliable to use a subtype B- backbone (as is currently the case with all available phenotypic assays) when assessing HIV-1 subtype C. In clinical practice, what this data suggests is that in the instance of subtype C, it is reliable for 95.8% of cases to use a B- backbone for phenotyping, once assay variability is to taken into account. Assay variability is especially important when FC values are close to the BCO's. Clinical decisions should not only be made merely according to the resistance call, but rather actual FC values should be considered. Caution should also be taken even when assessing resistance of non-B subtypes in a subtype B- backbone. This study indirectly suggests that the vircotype tool, which is built upon the Antivirogram database, is an equally reliable algorithm for genotyping subtype C samples. The Antivirogram® assay therefore remains an acceptable tool for phenotyping non-B GPRT amplicons.

## References

[1] WHOUNICEFUNAIDS (2010). Towards universal access. Scaling up priority HIV/AIDS interventions in the health sector.. Geneva, World Health Organization.

[2] Hamers RL, Derdelinckx I, van Vugt M, Stevens W, Rinke de Wit TR (2008). The status of HIV-1 resistance to antiretroviral drugs in sub-Saharan Africa.. Antiviral Therapy.

[3] Hunt GM, Coovadia A, Abrams EJ, Sherman G, Meyers T (2011). HIV-1 drug resistance at antiretroviral treatment initiation in children previously exposed to single-dose nevirapine.. AIDS Jul.

[4] Price M, Wallis C, Lakhi S, Karita E, Kamali A (2011). Transmitted HIV type 1 drug resistance among individuals with recent HIV infection in East and Southern Africa.. AIDS Res Hum Retroviruses Jan.

[5] Hamers RL, Wallis CL, Kityo C, Siwale M, Mandaliya K (2011). HIV-1 drug resistance in antiretroviral-naive individuals in sub-Saharan Africa after rollout of antiretroviral therapy: a multicentre observational study.. Lancet Infect Dis Jul.

[6] Hamers RL, Siwale M, Wallis CL, Labib M, van Hasselt R (2010). HIV-1 drug resistance mutations are present in six percent of persons initiating antiretroviral therapy in Lusaka, Zambia.. Journal of acquired immune deficiency syndromes (1999).

[7] Ndembi N, Hamers R, Sigaloff K, Lyagoba F, Magambo B (2011). Transmitted antiretroviral drug resistance among newly HIV-1 diagnosed young individuals in Kampala.. AIDS Apr.

[8] Hemelaar J, Gouws E, Ghys PD, Osmanov S (2011). Global trends in molecular epidemiology of HIV-1 during 2000–2007.. AIDS Mar.

[9] VanZyl G, VanderMerwe L, Claassen M, Zeier M, Preiser W (2011). Antiretroviral resistance patterns and factors associated with resistance in adult patients failing NNRTI-based regimens in the western cape, South Africa.. J Med Virol Oct;.

[10] Murphy RA, Sunpath H, Lu Z, Chelin N, Losina E (2010). Outcomes after virologic failure of first-line ART in South Africa.. AIDS.

[11] Virco BVBA (n.d.). Belgium.. http://www.vircolab.com/hiv-resistance-products/antivirogram.

[12] Hertogs K, de Béthune M, Miller V, Ivens T, Schel P (1998). A rapid method for simultaneous detection of phenotypic resistance to inhibitors of protease and reverse transcriptase in recombinant human immunodeficiency virus type 1 isolates from patients treated with antiretroviral drugs.. Antimicrob Agents Chemother Feb.

[13] Nauwelaers D, Houtte MV, Winters B, Steegen K, Baelen KV (2011). A Synthetic HIV-1 Subtype C Backbone Generates Comparable PR and RT Resistance Profiles to a Subtype B Backbone in a Recombinant Virus Assay.. PLoS ONE May.

[14] Wallis CL, Papathanasopoulos MA, Lakhi S, Karita E, Kaleebu P (2010). Affordable in-house antiretroviral drug resistance assay with good performance in non-subtype B HIV-1.. Journal of Virological Methods.

[15] Hamers R, Oyomopito R, Kityo C, Phanuphak P, Siwale M (2011). Cohort Profile: The PharmAccess African (PASER-M) and the TREAT Asia (TASER-M) Monitoring Studies to Evaluate Resistance-HIV drug resistance in sub-Saharan Africa and the Asia-Pacific.. Int J Epidemiol Apr.

[16] Rhee S-J, Gonzales M, Kantor R, Betts B, Ravela J (2003). Human immunodeficiency virus reverse transcriptase and protease sequence database.. Nucleic Acids Research.

[17] Virco B (n.d.). Virco Biological Cut-offs.. http://www.vircolab.com/hiv-resistance-products/vircotype-hiv-1/cut-offs-for-vircotype-hiv-1/biological-cut-offs.

[18] Vergara IA, Norambuena T, Ferrada E, Slater AW, Melo F (2008). StAR: a simple tool for the statistical comparison of ROC curves. BMC Bioinformatics 9: 265.. http://protein.bio.puc.cl/star.html.

[19] Johnson VA, Brun-Vézinet F, Clotet B, Günthard HF, Kuritzkes DR (2010). Update of the drug resistance mutations in HIV-1: December 2010.. Topics in HIV medicine : a publication of the International AIDS Society, USA.

[20] Choe S, Stawiski E, Parkin N (n.d.). Interpretation of Drug Susceptibility and Replication Capacity Results from Subtype C HIV-1 Protease/RT Is Not Influenced by the Subtype of the Resistance Test Vector..

[21] Qari SH, Respess R, Weinstock H, Beltrami EM, Hertogs K (2002). Comparative Analysis of Two Commercial Phenotypic Assays for Drug Susceptibility Testing of Human Immunodeficiency Virus Type 1.. J Clin Microbiol.

[22] VanHoutte M, Picchio G, Van Der Borght K, Pattery T, Lecocq P (2009). A comparison of HIV-1 drug susceptibility as provided by conventional phenotyping and by a phenotype prediction tool based on viral genotype.. J Med Virol Oct.

[23] Petropoulos C, Parkin N, Limoli K, Lie Y, Wrin T (2000). A novel phenotypic drug susceptibility assay for human immunodeficiency virus type 1.. Antimicrob Agents Chemother Apr.

[24] Steegen K, Bronze M, Van Craenenbroeck E, Winters B, Van der Borght K (2010). A comparative analysis of HIV drug resistance interpretation based on short reverse transcriptase sequences versus full sequences.. AIDS Res Ther Oct.

[25] Ariën K, Abraha A, Quiñones-Mateu M, Kestens L, Vanham G (2005). The replicative fitness of primary human immunodeficiency virus type 1 (HIV-1) group M, HIV-1 group O, and HIV-2 isolates.. J Virol Jul.

[26] Wang K, Samudrala R, Mittler JE (2004). Antivirogram or PhenoSense: a comparison of their reproducibility and an analysis of their correlation.. Antiviral Therapy Oct.

[27] Zhang J, Rhee S, Taylor J, Shafer RW (2005). Comparison of the Precision and Sensitivity of the Antivirogram and PhenoSense HIV Drug Susceptibility Assays.. J Acquir Immune Defic Syndr.

